# Editorial: Antioxidants and inflammatory immune-related diseases

**DOI:** 10.3389/fimmu.2024.1476887

**Published:** 2024-08-19

**Authors:** Pengfei Xu, Zunnan Huang, Yanyong Xu, Huanxiang Liu, Yuyu Liu, Ling Wang

**Affiliations:** ^1^ Department of Hepatobiliary and Pancreatic Surgery, Zhongnan Hospital of Wuhan University, Hubei Provincial Key Laboratory of Developmentally Originated Disease, School of Pharmaceutical Sciences, Wuhan University, Wuhan, China; ^2^ Key Laboratory of Computer-Aided Drug Design of Dongguan City, School of Pharmacy, Guangdong Medical University, Dongguan, China; ^3^ Key Laboratory of Metabolism and Molecular Medicine of the Ministry of Education, Department of Pathology of School of Basic Medical Sciences, Frontier Innovation Center, Fudan University, Shanghai, China; ^4^ Faculty of Applied Sciences, Macao Polytechnic University, Macao, Macao SAR, China; ^5^ School of Ocean and Tropical Medicine, Guangdong Medical University, Zhanjiang, China; ^6^ Guangdong Provincial Key Laboratory of Fermentation and Enzyme Engineering, Joint International Research Laboratory of Synthetic Biology and Medicine, Guangdong Provincial Engineering and Technology Research Center of Biopharmaceuticals, School of Biology and Biological Engineering, South China University of Technology, Guangzhou, China

**Keywords:** inflammation, autoimmune, reactive oxygen species, damage-associated molecular patterns, antioxidants

The interplay between antioxidants and inflammatory immune-related diseases has gained significant attention in recent years. Antioxidants, known for combating oxidative stress, have emerged as potential modulators of inflammation, a process intricately linked to various immune-related conditions (1). Understanding the role of antioxidants in managing these diseases holds the promise for advancing preventive and therapeutic strategies. This editorial explores the multifaceted connection between antioxidants and inflammation, highlighting their potential health implications.

Inflammation, the body’s natural response to injury or infection, is crucial for maintaining homeostasis (2). However, chronic inflammation can lead to the development and progression of numerous immune-related diseases, such as rheumatoid arthritis, inflammatory bowel disease (IBD), and certain cancers (3). The complex cellular and molecular mechanisms of chronic inflammation highlight its significant impact on overall health.

The relationship between antioxidants and inflammatory immune-related diseases centers on oxidative stress. Free radicals, byproducts of normal cellular processes, can cause oxidative damage, triggering and sustaining inflammation (4). Antioxidants neutralize free radicals, thereby counteracting oxidative stress and potentially modulating the inflammatory response. This modulation may alleviate symptoms and slow the progression of inflammatory diseases, presenting a compelling area for exploration.

Integrating antioxidant-rich foods into one’s daily diet holds significant practical value for holistic health (5). A balanced and diverse diet, rich in antioxidants like vitamin C, vitamin E, beta-carotene, and selenium, can promote overall well-being and potentially mitigate chronic inflammation (6). These antioxidants, found in fruits, vegetables, nuts, and seeds, provide essential nutrients and support the body’s defense against oxidative stress and inflammation (7). However, antioxidants should not be regarded as standalone treatments for inflammatory immune-related diseases. Personalized advice from healthcare professionals is essential in developing effective preventive and therapeutic strategies.

Research into the potential benefits of antioxidants in managing inflammation has yielded compelling evidence. Studies have elucidated the mechanisms through which antioxidants exert their anti-inflammatory effects, offering insights into their therapeutic implications (8–10). While some studies report positive associations between antioxidant intake and reduced inflammatory markers, others yield inconclusive findings, highlighting the complexity of this field (11–13). This Research Topic gathers researchers worldwide to explore novel therapeutic avenues for managing inflammatory diseases, including bacterial infections, arthritis, IBD, and gastric cancer.

Liu et al. summarized the biological mechanisms and potential applications of cell migration–inducing protein (CEMIP) in various diseases. Also known as KIAA1199, CEMIP is a member of the hyaluronidase family and plays a key role in degrading hyaluronic acid (HA) and remodeling the extracellular matrix (14). They highlighted CEMIP’s involvement in promoting tumor cell proliferation, invasion, and adhesion, as well as its role in bacterial infections and arthritis. The review focused on CEMIP’s pathological mechanisms, including inhibiting cell apoptosis, promoting HA degradation, inducing inflammatory responses, modulating the cellular microenvironment, and regulating tissue fibrosis. Additionally, the review provided insights into potential diagnostic and treatment strategies targeting CEMIP, emphasizing its diverse roles and promising applications in various diseases.

Inflammatory-erosive arthritis is worsened by dysfunction in joint-draining popliteal lymphatic vessels (PLVs) (15). Synovial mast cells are known to be pro-inflammatory in rheumatoid arthritis (16). Peng et al. investigated the impact of mast cells on joint-draining PLVs and the progression of inflammatory-erosive arthritis in tumor necrosis factor transgenic (TNF-tg) mice. Using various imaging and analytical techniques, the study revealed that mast cells are crucial for normal lymphatic function and arthritis progression. Findings indicated that mast cells’ activation and degranulation contribute to inflammatory-erosive arthritis. Genetic ablation and pharmacological inhibition of mast cells exacerbated TNF-induced arthritis and decreased lymphatic clearance. The study suggested that peri-PLV mast cells have an inflammatory role, while intra-PLV mast cells play a homeostatic role, and their loss exacerbates arthritis due to defects in joint-draining lymphatics. These results highlight the need for further investigation into the specific cellular mechanisms underlying these effects.

Gastric precancerous lesions (GPL) are a significant global health concern due to their potential to progress to gastric cancer (GC) (17). Zhang et al. reviewed the role of chronic inflammation in GPL progression, emphasizing its implications for disease advancement and therapeutic applications. The review highlighted helicobacter pylori (H. pylori) as a mediator of the inflammatory microenvironment and a key driver of GC progression. It also discussed the involvement of immune cells and the regulation of inflammatory molecules in GPL, as well as the potential for targeting inflammatory pathways for treatment. Additionally, the review highlighted the promising effects of traditional Chinese Medicine and natural antioxidants in suppressing or reversing GPL progression. The paper concluded by proposing future perspectives and innovative therapeutic approaches in this field.

Zou et al. utilized Mendelian randomization (MR) to investigate the causal relationship between circulating antioxidants and IBD. Instrumental variables for circulating antioxidants were identified from published studies, and outcome data were derived from two genome-wide association study (GWAS) databases. The MR analysis and meta-analysis indicated that increased circulating levels of retinol were significantly associated with a reduced risk of ulcerative colitis (UC). The study also found suggestive evidence of a negative association between retinol levels and IBD. However, no other causal relationships were identified. The conclusion underscores the robust evidence linking retinol levels to a decreased risk of UC and suggests the necessity for further MR studies with additional instrumental variables for circulating antioxidants to validate these findings.

Understanding the potential of antioxidants has significant implications for human health. This Research Topic provides an open-access platform for reviews and original research on how antioxidants can treat inflammatory and immune-related diseases. By modulating inflammation and promoting overall well-being, antioxidants present a promising path for developing preventive and therapeutic strategies. Gaining a comprehensive understanding of inflammation’s complexities is essential, as is a commitment to evidence-based health and wellness approaches. Continued exploration of the relationship between antioxidants and inflammation is crucial, paving the way for a healthier future.
